# Stress-Driven Generation of Continuous Fibrous Material Paths for Additive Manufacturing: Numerical Assessment and Manufacturing Feasibility

**DOI:** 10.3390/ma19091868

**Published:** 2026-05-01

**Authors:** Andrea Sellitto, Aniello Riccio

**Affiliations:** Department of Engineering, University of Campania Luigi Vanvitelli, Via Roma 29, 81031 Aversa, CE, Italy; aniello.riccio@unicampania.it

**Keywords:** continuous fibre additive manufacturing, fibre orientation, principal stress directions, streamline-based path planning, composite materials, anisotropy, finite element analysis, toolpath generation

## Abstract

**Highlights:**

- Semi-automated generation of manufacturable fibre paths from stress-aligned streamlines.
- Continuous and directly 3D-printable fibre toolpaths.
- Increased structural performances compared to conventional unidirectional layouts.
- Enables efficient exploitation of anisotropy in continuous fibre additive manufacturing.
- Translates FEM-based stress information into executable fibre trajectories.

**Abstract:**

This work presents a methodology for the generation of continuous fibre trajectories based on principal stress directions in continuous fibre-reinforced additive manufacturing (CFAM). The material system considered consists of continuous carbon fibre (CCF-1.5K) embedded in a CFC-PA thermoplastic matrix. CFAM enables the deposition of fibres along tailored paths, allowing improved alignment with the load direction, compared to traditional composite manufacturing. In this way, the strong anisotropy of composite materials, typically considered a limitation, is exploited as a design opportunity by aligning fibres with the structural load paths. The proposed approach combines finite element analysis with a path generation procedure, including the computation of principal stress directions, the extraction of streamlines of the principal stress field, and a dedicated post-processing stage aimed at obtaining continuous and manufacturable fibre layouts. The effectiveness of the method is assessed through a finite element-based comparison with conventional fibre configurations, showing an increase in global stiffness of approximately 20% with respect to the best-performing unidirectional layout. In addition, the feasibility of the generated trajectories is demonstrated through printing tests performed on a continuous fibre additive manufacturing system. The results confirm that the proposed methodology enables the generation of physically realizable fibre paths while improving structural performance.

## 1. Introduction

Carbon fibre-reinforced composites are widely used in structural applications due to their high specific stiffness and strength compared to conventional metallic alloys. However, unlike metals, these materials exhibit a strongly anisotropic behaviour, with mechanical properties that depend on the orientation of the reinforcing fibres [1].

While this anisotropy represents a design challenge in traditional manufacturing processes, it can also be exploited as an opportunity when appropriate fibre orientations are adopted. In this context, additive manufacturing technologies provide a unique advantage, since they enable a high level of control over the material placement and fibre trajectories within a component [2,3,4]. Recent studies have also highlighted the potential of additive manufacturing for advanced composite structures, enabling complex geometries and tailored material distributions not achievable with conventional techniques [5,6].

Continuous fibre-reinforced additive manufacturing (CFAM) allows combining the geometric freedom of layer-by-layer fabrication with the capability of locally tailoring fibre orientations. This allows fibre alignment with the load paths of the structure, thereby improving structural efficiency and reducing the limitations associated with uniform fibre layouts [7,8,9]. Recent contributions can be found in the literature, exploring the integration of design and manufacturing constraints in CFAM, highlighting the importance of continuous path planning and process-aware optimization strategies [10].

In this work, a continuous fibre co-extrusion process based on fused filament fabrication (FFF) is considered, in which continuous carbon fibre (CCF-1.5K) is deposited together with a CFC-PA thermoplastic matrix. In such systems, the mechanical response is governed by the anisotropic behaviour resulting from fibre–matrix interaction and load transfer mechanisms. In the context of CFAM, the ability to control fibre orientation during deposition therefore represents a key factor in tailoring the structural performance of the component under different loading conditions.

Although fibre positioning strategies based on stress distributions have been extensively studied in previous research [11,12,13,14], including load-dependent path planning and stress-vector-tracing approaches for continuous fibre deposition [15], the translation of stress fields into fibre trajectories that can be directly manufactured remains an issue that has been only partially addressed and often requires manual post-processing. This work contributes to this field by proposing a practical workflow for generating continuous fibre paths suitable for additive manufacturing, with reduced manual intervention. Indeed, the generation of continuous and manufacturable fibre paths remains a challenging task [16,17]. In particular, stress-based trajectories are often discontinuous or geometrically complex, making them difficult to directly translate into printable toolpaths. Moreover, additional strategies are required to ensure full coverage of the domain while preserving consistency with the underlying stress field. Recent works have also investigated concurrent optimization strategies and smooth fibre orientation constraints; however, challenges related to trajectory continuity and manufacturability remain open [18,19].

In this work, a methodology for the generation of continuous fibre trajectories based on principal stress directions is proposed. The approach combines finite element analysis with a path generation procedure, including streamline extraction and a dedicated post-processing stage aimed at producing continuous and manufacturable fibre layouts.

The effectiveness of the proposed method is assessed through a finite element-based comparison with a reference configuration, showing an improvement in global stiffness. In addition, the feasibility of the generated trajectories is demonstrated through printing tests performed using a continuous fibre additive manufacturing system.

The main objective of this work is to develop and validate a stress-driven methodology able to define continuous and manufacturable fibre trajectories in CFAM. In particular, the study aims to generate fibre paths aligned with the principal stress directions, while ensuring the continuity and manufacturability of the resulting trajectories, and to assess the structural performance of the proposed approach in comparison with conventional fibre layouts.

The paper is organized as follows. Section 2 introduces the theoretical background. Section 3 describes the proposed methodology. Section 4 presents the numerical assessment. Section 5 discusses the manufacturing feasibility.

## 2. Theoretical Background

The mechanical behaviour of fibre-reinforced structures is strongly influenced by the orientation of the reinforcing fibres with respect to the applied load. In continuous fibre additive manufacturing, this dependency can be used by aligning fibre trajectories with the principal stress directions, consequently improving the structure load-carrying capability [11,20].

Under plane stress conditions, the local stress state at a specific location can be described by the Cauchy stress tensor:

(1) $$\sigma =\left[\begin{array}{cc} \sigma_{xx} & \tau_{xy}\\ \tau_{xy} & \sigma_{yy} \end{array}\right].$$

Starting from Equation (1), the principal stresses *σ*_1_ and *σ*_2_ can be derived as the eigenvalues of the stress tensor:

(2) $$\sigma_{1,2}=\frac{\sigma_{xx}+\sigma_{yy}}{2}\pm \sqrt{{\left(\frac{\sigma_{xx}-\sigma_{yy}}{2}\right)}^{2}+\tau_{xy}^{2}}.$$

The associated eigenvectors define the principal stress directions, which correspond to orientations where the shear stress component is equal to zero. These directions represent the natural load paths within the structure and are often referred to as stress trajectories or load paths [15,21,22,23].

In anisotropic composite materials, the stiffness is highly dependent on the material orientation with respect to the applied load. In a unidirectional composite lamina, the longitudinal modulus *E*_1_ is significantly higher than the transverse modulus *E*_2_. Therefore, the alignment of the fibres with respect to the direction of maximum principal stress *σ*_1_ allows the structure to take advantage of the higher stiffness and strength along the fibre direction [24,25,26].

From a structural perspective, aligning the fibres with the directions of the principal stresses reduces shear components and allows for efficient load transfer within the material. In this work, the principal stress directions are computed from a preliminary finite element analysis and used to define a continuous vector field over the domain. Considering the unit eigenvector *v*_1_(x), associated with the maximum principal stress *σ*_1_ at location x, the fibre orientation field *d*(*x*) can be defined as:

(3) $$d(x)=v_{1}\left(x\right).$$

Fibre trajectories are obtained as streamlines of this vector field. From a mathematical point of view, streamlines are curves *x*(*s*) whose tangent direction aligns with the local fibre orientation:

(4) $$\frac{dx\left(s\right)}{ds}=d\left(x\left(s\right)\right),$$

where $\frac{dx\left(s\right)}{ds}$ is the derivative of the streamline *x*(*s*), representing its tangent direction, while *d*(*x*(*s*)) is the fibre orientation field (Equation (3)) at the current position *s* along the streamline. Hence, the directional field is evaluated over a discretized representation of the domain, and streamline trajectories are generated numerically based on this field.

The resulting set of curves represents fibre paths aligned with the principal stress directions, effectively approximating the load transmission paths within the structure [13]. However, due to discretization and local variations in the stress field, the raw trajectories may exhibit discontinuities or excessive curvature. Therefore, additional smoothing and post-processing operations are required to obtain manufacturable fibre paths, as described in the following section.

## 3. Proposed Stress-Driven Toolpath Generation Method

**Figure 1:**
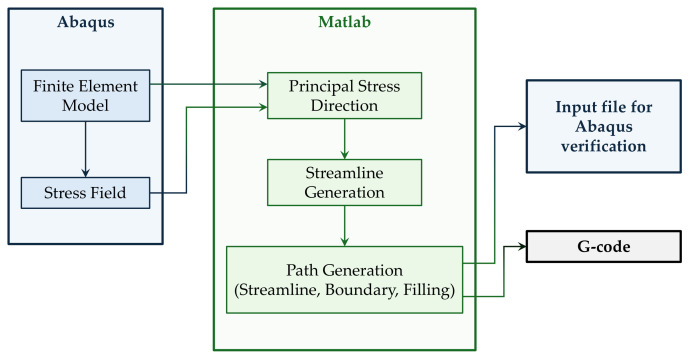
Workflow of the proposed procedure.

In this section, the proposed method for generating fibre trajectories aligned with the principal stress directions is presented. The workflow, implemented through a Matlab (R2024b) in-house tool, is based on a preliminary finite element analysis, followed by the extraction of the stress field, the computation of principal stress directions, and the generation of fibre paths based on these directions. Indeed, the definition of continuous deposition paths represents a key aspect in continuous fibre additive manufacturing, and has been addressed in different contexts, including path-driven design strategies for complex geometries [27]. In particular, stress-aligned trajectories are first obtained based on selected streamlines, and then complemented with additional paths along the boundaries and concentric filling patterns to ensure geometric fidelity and complete filling of the domain. Finally, the obtained trajectories are post-processed in order to ensure their manufacturability and converted into printable toolpaths. This step is particularly relevant, since the generation of continuous and smooth fibre paths remains a well-known challenge in stress-driven approaches [28]. An overview of the proposed workflow is shown in Figure 1.

The following subsections describe each step of the procedure in detail.

### 3.1. Case Study Definition

The investigated case study consists of a two-dimensional rectangular holed plate subjected to tensile loading. This specific test case has been selected as a representative benchmark problem due to stress concentrations around the hole.

**Figure 2:**
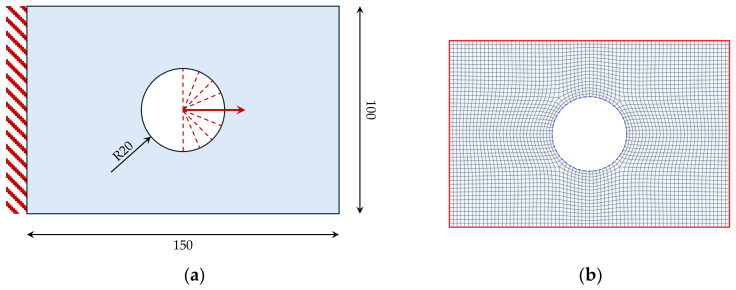
(**a**) Geometry (dimension in mm) and boundary conditions; (**b**) Finite Element Model.

A finite element model is adopted to compute the stress distribution within the domain. The preliminary analyses are performed in the Abaqus environment. A sufficiently refined mesh is used in order to accurately capture the stress gradients, especially in the proximity of the hole. Figure 2a shows the geometry and the boundary conditions, while the finite element model is shown in Figure 2b. The domain has been discretized using 4-node shell elements with a reduced integration scheme (S4R). A preliminary mesh sensitivity analysis was carried out to assess the influence of the discretization on the resulting fibre trajectories, as discussed in the following section.

According to Figure 2a, one side of the panel has been fixed, while the load has been distributed along the opposite half of the internal hole.

**Figure 3:**
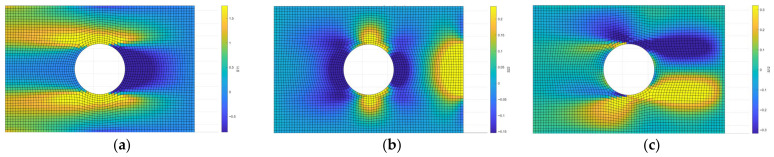
Stress components: (**a**) *σ_xx_*; (**b**) *σ_yy_*; (**c**) *τ_xy_*.

The material is assumed to be linear, elastic and isotropic. This choice is motivated by the need to obtain a stress field that is not biassed by predefined material orientations, allowing the principal stress directions to naturally follow the applied loading conditions. Since the scope of the proposed method is to extract the principal stress directions rather than their magnitude, a relatively low load (10 N) is applied in the finite element analysis. Indeed, under linear elastic conditions, the stress field is proportional to the applied load, while no variation occurs in the principal stress directions. Therefore, the specific value of the applied load does not affect the resulting fibre orientations. This also explains the relatively low magnitude of the computed stress values, shown in Figure 3, which are not relevant for the purposes of this study.

Both the finite element model and the resulting stress field, expressed in terms of *σ_xx_*, *σ_yy_* and *τ_xy_*, are then used as input for the subsequent steps of the workflow, implemented in the custom Matlab-based tool.

### 3.2. Principal Stress Direction Field

The second step of the proposed workflow consists of extracting the principal stress directions from the stress field obtained through the finite element analysis.

Starting from the stress components *σ_xx_*, *σ_yy_* and *τ_xy_*, the principal stresses and their associated directions are computed at discrete location within the domain. The principal stress values are obtained according to Equation (2), while the corresponding directions are used to define the fibre orientation field *d*(*x*) as introduced in Equation (3). In particular, the unit eigenvector *v*_1_(x), associated with the maximum principal stress *σ*_1_, is evaluated and used to define the local fibre orientation.

**Figure 4:**
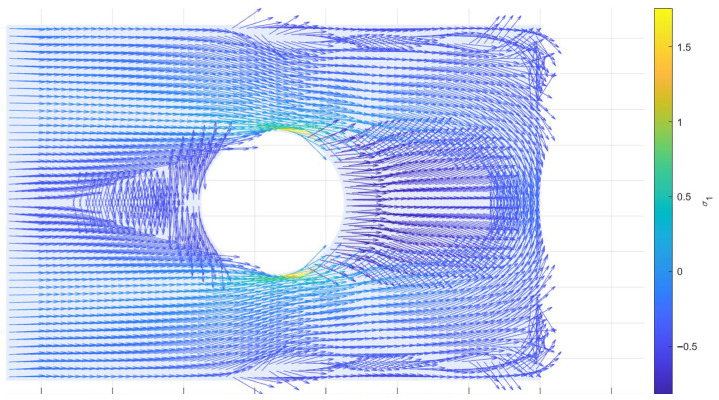
Principal stress directions.

Since the stress field is obtained from a discrete finite element model, the principal directions are initially defined at discrete locations, corresponding to the integration point of each element, as shown in Figure 4. In order to enable streamline generation, these directions are subsequently mapped onto a discretized representation of the domain, resulting in a directional field suitable for trajectory definition.

### 3.3. Streamline Generation

The third step of the proposed workflow focuses on the generation of continuous fibre trajectories, starting from the previously defined directional field.

In order to enable streamline definition, the element-wise fibre directions are mapped onto a regular Cartesian grid covering the domain. Each grid point is assigned the direction associated with the element to which it belongs, resulting in a grid-based directional field.

**Figure 5:**
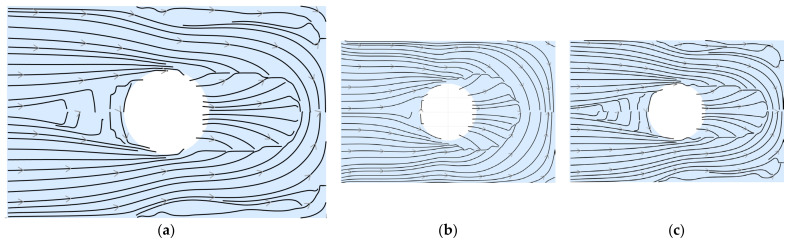
Streamlines generated from the principal stress directions: (**a**) reference mesh; (**b**) coarser mesh; (**c**) finer mesh.

Based on this field, streamline trajectories are generated within the Matlab environment using built-in streamline integration functions. These curves, shown in Figure 5a, follow the local orientation of the directional field and represent continuous paths aligned with the principal stress directions. It is worth noting that, due to the adopted sign convention for the principal directions, the resulting streamlines show a consistent global orientation across the domain.

A preliminary mesh sensitivity analysis was carried out to evaluate the influence of discretization on the extracted streamlines. Figure 5b,c show the fibre trajectories obtained using coarser and finer meshes, corresponding respectively to approximately 1.5 times larger and smaller element sizes compared to the reference mesh. The comparison suggests that the overall patterns and orientations of the streamlines remain consistent, with only slight local variations. Based on this analysis, the reference mesh was selected as a compromise between computational cost and accuracy, since further refinement did not lead to significant changes in the resulting fibre trajectories.

The resulting streamlines constitute the first set of stress-aligned trajectories and provide a geometrically continuous representation of the load transmission paths within the structure. However, due to the discrete nature of the underlying finite element model and the grid-based representation of the directional field, the generated trajectories may not form a complete or fully connected description of the load paths. In particular, some streamlines may terminate prematurely or fail to capture complex stress patterns in regions characterized by strong stress gradients, such as around geometric discontinuities.

For this reason, a dedicated post-processing stage is required in order to refine and complete the set of trajectories, as described in the following subsection.

### 3.4. Path Generation

The streamline generation step provides a first set of stress-aligned trajectories. However, these paths cannot be directly used to define printable paths, since they may be incomplete, discontinuous or geometrically irregular. Therefore, a post-processing stage is required in order to refine and complete the fibre layout.

A first operation consists of analyzing the generated streamlines in terms of both geometric and mechanical properties. For each trajectory, quantities such as total length, mean principal stress and maximum principal stress are evaluated. These indicators are used to support the identification of the most relevant trajectories within the set of generated streamlines.

It is worth noting that these metrics are not intended to define an optimal selection criterion. Instead, they provide a practical support for the identification of meaningful load paths, and the final selection may involve user-guided selection. This approach allows maintaining flexibility in the path selection process, which is particularly relevant when additional constraints related to manufacturability must be considered.

In addition, the initial set of streamlines may not form a connected or complete representation of the load paths. In order to address this issue, neighbouring trajectories can be combined to improve continuity and capture a more complete behaviour of the underlying stress patterns. Moreover, prior to the generation of fibre paths, a smoothing operation is applied to the selected trajectories in order to reduce local irregularities and curvature variations. This step is performed at the streamline level to ensure that the subsequent path generation process produces geometrically consistent and non-overlapping fibres, and it is particularly relevant in regions characterized by high curvature or complex geometrical features, where raw streamlines may exhibit abrupt changes.

**Figure 6:**
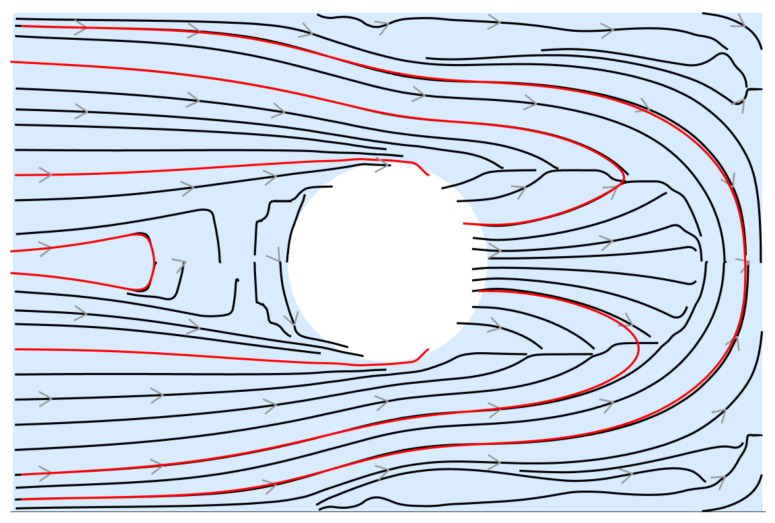
Selected streamlines (in red).

This choice is motivated by manufacturability considerations, as applying smoothing after path generation may lead to local intersections or violations of minimum spacing constraints. The selected streamlines for the considered case study are shown in Figure 6.

Once a consistent set of primary trajectories has been defined, boundary-following paths are generated along the edges of the geometry. This operation is performed before the generation of internal paths, as the stress-aligned trajectories extend up to the domain boundaries; hence, defining boundary paths in advance ensures that sufficient space is reserved to the fibre placement while preserving the geometric integrity of the component. Moreover, the number of boundary layers is defined as a user-controlled parameter and can be adjusted depending on the desired structural and manufacturing requirements. In particular, this parameter may also be set to zero, allowing the generation of fibre layouts without boundary reinforcement.

Subsequently, fibre paths are generated starting from the selected and smoothed streamlines. Each trajectory is used as a reference and iteratively offset by a distance corresponding to the fibre width. The process is repeated until a stopping condition is reached. In particular, path generation is interrupted when the offset curve completely exits the domain, intersects previously generated fibres, or becomes geometrically discontinuous.

Finally, the remaining regions of the domain not covered by the boundary-following or the stress-aligned paths are filled using additional trajectories. For each uncovered region, boundary-following paths are generated in the form of closed loops, obtained by iteratively offsetting the region boundaries. At each iteration, the generated path reduces the size of the uncovered region, which is then updated accordingly. The process is repeated until the residual areas are completely filled or no further valid paths can be generated. In this way, a set of contour-parallel trajectories is obtained, which ensure an effective filling of the domain while maintaining geometric consistency with the previously generated fibre paths.

**Figure 7:**
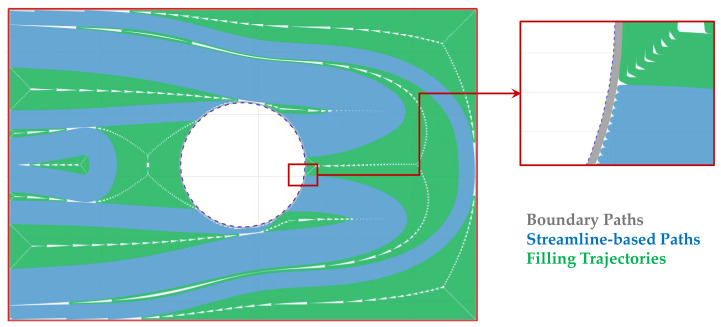
Generated trajectories: (grey) boundary paths, (blue) streamline-based paths, and (green) filling trajectories.

The outcome of this process is a continuous and printable set of toolpaths, which is used for the generation of the printing instructions. In Figure 7, the generated trajectories are shown, where boundary paths are represented in grey, streamline-based paths in blue, and filling trajectories in green.

## 4. Finite Element-Based Assessment

### 4.1. Element-Wise Material Modelling

In order to numerically assess the structural response of the generated fibre layout, the optimized trajectories are mapped back onto the finite element mesh of the reference case study.

For each element, the local fibre orientations and the corresponding fibre volume fractions are evaluated on the basis of the fibre segments intersecting the element. These quantities are then used to compute an equivalent orthotropic compliance matrix representative of the local material behaviour of each element.

It is worth noting that the total fibre volume fraction within a given element may be lower than 100%, as some regions may remain partially uncovered by the generated trajectories. In these cases, the remaining fraction is assumed to be filled by matrix material only. More specifically, the uncovered portion is assigned the transverse matrix-dominated property of the composite filament.

**Figure 8:**
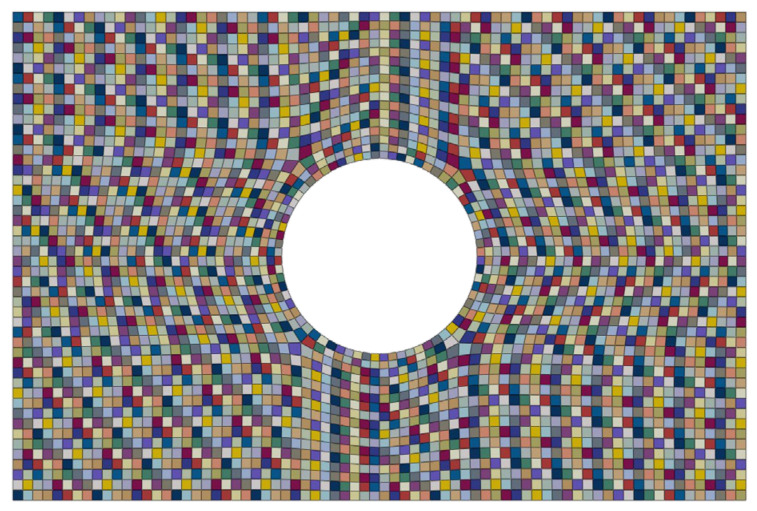
Element-wise distribution of the equivalent orthotropic material properties assigned to the finite element model of the optimized configuration.

As a result, an equivalent orthotropic material is obtained for each element of the mesh. Starting from the original Abaqus input file of the case study, a new input file is generated directly by the Matlab-based tool by assigning element-wise material properties consistent with the local fibre distribution and orientation. The resulting distribution of the assigned material properties is shown in Figure 8.

### 4.2. Structural Performance Comparison

The structural performance of the proposed fibre layout is evaluated by comparing the optimized configuration with a set of conventional ones.

The conventional configurations consist of a unidirectional fibre layout aligned with the loading direction (0°) and normal to it (90°), as well as cross-ply configurations, specifically 0°/90° and ±45°. These configurations can be considered as conventional design and are used as a baseline for comparison.

All models are analyzed using the same boundary conditions and loading scheme. The material properties are kept constant across all simulations in order to isolate the influence of fibre layout on the structural response. The comparison is carried out in terms of global stiffness, defined as the ratio between the applied load and the resulting displacement, as a representative indicator for the present study. It should be noted that the numerical analysis is intended to provide a consistent relative comparison between different fibre layout strategies, rather than an absolute prediction of the mechanical response. The results of the comparisons are reported in Table 1.

According to Table 1, among the considered cases, the unidirectional 0° configuration is characterized by the highest stiffness and is therefore taken as the reference for comparison. The reference configuration exhibits a stiffness equal to 4507.63 N/mm, while the optimized configuration reaches 5437.41 N/mm. This corresponds to a stiffness increase of approximately 20.6%. This improvement, obtained with respect to the best-performing conventional configuration, shows the effectiveness of the proposed approach in improving the structural response by tailoring the fibre orientation according to the stress distribution.

The obtained results are consistent with previous studies on stress-based fibre placement strategies, which have demonstrated that aligning fibres with the directions of the principal stresses improves structural performance compared to conventional layouts [11,12,13,15]. Similar approaches based on load-path-oriented design and stress vector tracing have demonstrated improved load-carrying capability by following the natural stress paths within the structure. However, most existing work focuses primarily on defining fibre orientation fields or path-planning strategies, while aspects related to path continuity and manufacturability remain challenging, especially in the case of complex geometries or high stress gradients [27,28].

In this context, this work contributes by providing a comprehensive workflow that integrates stress analysis, streamline extraction and path post-processing, enabling the generation of continuous and printable fibre trajectories with minimal manual intervention. Compared to the optimization-based approaches recently proposed in the literature [29], the method maintains a relatively simple and computationally efficient formulation, ensuring practical applicability in a manufacturing-oriented context.

## 5. Manufacturing Feasibility

In order to assess the practical applicability of the proposed methodology, a set of printing tests is performed based on the generated toolpaths. The printing tests are carried out using an Anisoprint Composer A3 system, (Anisoprint SARL, Esch-sur-Alzette, Luxembourg) specifically designed for continuous fibre additive manufacturing. The printing system is based on a co-extrusion process, in which a continuous dry carbon fibre is combined with a thermoplastic filament within the same nozzle during deposition, enabling the in situ impregnation of the fibre.

The fibre trajectories obtained from the workflow are converted by the Matlab procedure into G-code and used to fabricate representative specimens. The aim of this step is not to evaluate the mechanical performance of the printed parts, but rather to demonstrate the feasibility of the proposed path-generation strategy.

**Figure 9:**
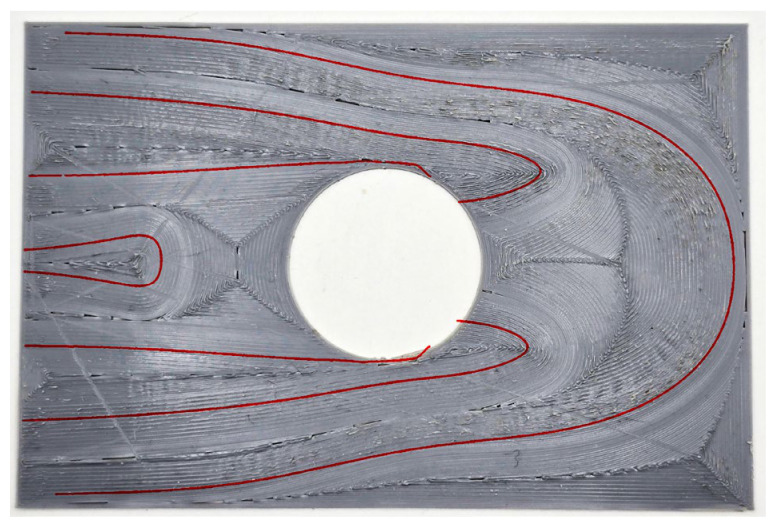
PLA specimen used to validate the generated toolpaths superimposed with the selected streamlines (in red).

A first specimen is produced using standard PLA in order to validate the correctness of the generated toolpaths and the overall printing sequence. The resulting part, shown in Figure 9, confirms that the generated paths can be successfully translated into executable printing instructions.

Figure 9 shows the printed specimen superimposed to the selected streamline used to define the fibre trajectories. It should be noted that due to the adopted continuous deposition strategy, adjacent paths are printed in alternating directions. Therefore, Figure 9 is focused on the orientation of the fibres rather than the deposition sequence.

**Figure 10:**
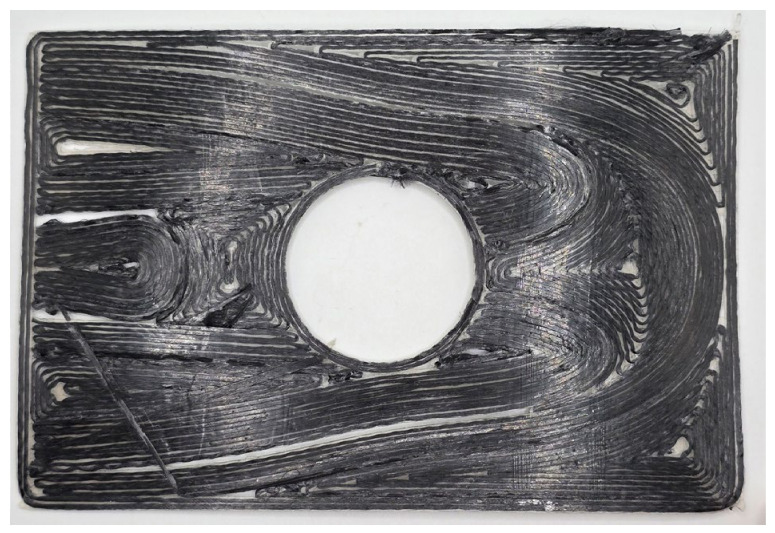
Single-layer continuous fibre specimen showing the alignment of the printed fibres with the generated trajectories. For visualization purposes, the fibre spacing is increased to 1 mm, compared to the actual value of 0.7 mm used in the manufacturing process.

In addition, a specimen is fabricated using continuous fibre reinforcement. In this case, a single-layer deposition is considered in order to clearly highlight the orientation of the fibres along the generated trajectories. It is worth noting that the spacing between adjacent fibres in the printed sample is intentionally increased with respect to a fully dense configuration (from 0.7 mm to 1 mm) to allow for a clearer visualization of the fibre paths and to facilitate the qualitative assessment of the alignment between the printed fibres and the expected stress directions. The printed specimen, shown in Figure 10, confirms that the proposed methodology can be effectively used with continuous fibre additive manufacturing processes and translated into physically manufacturable fibre layouts.

## 6. Conclusions

In this work, a methodology for the generation of continuous carbon fibre trajectories based on principal stress directions in CFAM has been presented. The proposed approach combines finite element analysis with a path generation procedure to obtain fibre layouts aligned with the load paths of the structure.

The workflow integrates stress analysis, streamline extraction, and a dedicated post-processing stage aimed at producing continuous and manufacturable fibre paths. The introduction of boundary paths, streamline-based trajectories, and contour-based filling strategies allows a complete and consistent fibre layout.

The effectiveness of the proposed approach has been assessed through a finite element-based comparison with conventional configurations, showing an increase in global stiffness of approximately 20%. The feasibility of the generated trajectories has been further demonstrated through printing tests, confirming their translation into executable toolpaths and physically realizable fibre layouts.

The results confirm that the objectives of the study have been achieved, demonstrating the capability of the proposed methodology to generate continuous and manufacturable fibre trajectories and to improve structural performance compared to conventional layouts.

It should be noted that the stress field is initially computed assuming isotropic material behaviour. Moreover, the present study does not explicitly account for potential manufacturing-induced effects, such as fibre misalignment, defects related to the deposition process, or limitations associated with minimum curvature and steering constraints. In particular, local deviations from the ideal fibre orientation may occur during printing, especially in regions characterized by high curvature or complex geometrical features. These aspects may influence the actual mechanical performance of the manufactured component and are not captured in the current numerical framework.

However, it should be emphasized that the primary objective of the proposed methodology is not the detailed prediction of the mechanical response, but rather the generation of continuous and manufacturable fibre trajectories that can be directly translated into executable toolpaths. In this context, the numerical model is employed as a supporting tool for the extraction of stress-driven orientations, while the main contribution lies in the development of a practical workflow for path generation compatible with continuous fibre additive manufacturing processes.

Future developments will consider iterative approaches to update the stress field based on the optimized fibre orientations, as well as the integration of manufacturing constraints and process-induced effects within the modelling framework, and experimental validation to further assess the mechanical performance of the proposed methodology.

## Figures and Tables

**Table 1:** Global stiffness of the reference and optimized configurations.

Configuration	Stiffness [N/mm]	Normalized Stiffness
0° Conventional Configuration	4507.63	1.00
90° Conventional Configuration	913.90	0.20
0°/90° Conventional Configuration	2388.39	0.53
45°/−45° Conventional Configuration	1717.63	0.38
Optimized Configuration	5437.41	1.21

## Data Availability

The original contributions presented in this study are included in the article. Further inquiries can be directed to the corresponding author.

## Abbreviations

The following abbreviations are used in this manuscript:

AM	Additive Manufacturing
CFAM	Continuous Fibre Additive Manufacturing
FEM	Finite Element Method
FFF	Fused Filament Fabrication
PLA	Polylactide
